# A Meta-Analysis of *P2X7* Gene-762T/C Polymorphism and Pulmonary Tuberculosis Susceptibility

**DOI:** 10.1371/journal.pone.0096359

**Published:** 2014-05-08

**Authors:** Lingling Yi, Dan Cheng, Huimin Shi, Xiaorong Huo, Kan Zhang, Guohua Zhen

**Affiliations:** Department of Respiratory and Critical Care Medicine, Tongji Hospital, Tongji Medical College, Huazhong University of Science and Technology, and Key Laboratory of Respiratory Diseases, Ministry of Health, Wuhan, Hubei, China; Tor Vergata University of Rome, Italy

## Abstract

**Aim:**

We performed a comprehensive meta-analysis to determine the association between *P2X7* -762T/C polymorphism and pulmonary tuberculosis susceptibility.

**Methodology:**

Based on comprehensive searches of the PubMed, SCI, Elsevier, China National Knowledge Infrastructure (CNKI) and Wanfang Database, we identified eligible studies about the association between *P2X7* -762T/C polymorphism and pulmonary tuberculosis risk. Pooled odds ratio (ORs) and 95% confidence intervals (95%CIs) were calculated in random-effects model.

**Results:**

A total of 2207 tuberculosis cases and 2220 controls in 8 case-control studies were included in this meta-analysis. Allele model (C vs. T: p = 0.15; OR = 0.83, 95% CI = 0.65–1.07), homozygous model (CC vs. TT: p = 0.23; OR = 0.73, 95% CI = 0.44 to 1.22), and heterozygous model (CT vs. TT: p = 0.57; OR = 0.92, 95% CI = 0.68 to 1.24) did not show increased risk of developing pulmonary tuberculosis. Similarly, dominant model (CC+CT vs. TT: p = 0.32; OR = 0.84, 95% CI = 0.59 to 1.19) and recessive model (CC vs. CT+TT: p = 0.08; OR = 0.77, 95% CI = 0.57 to 1.04) failed to show increased risk of developing pulmonary tuberculosis. Subgroup analysis by ethnicity did not detect any significant association between *P2X7*–762T/C polymorphism and pulmonary tuberculosis susceptibility.

**Conclusions:**

*P2X7* -762T/C gene polymorphism is not associated with pulmonary tuberculosis susceptibility.

## Introduction

Tuberculosis is caused by the bacillus *Mycobacterium tuberculosis* and remains a major challenge to global public health [1]. According to the report of World Health Organization, there were an estimated 8.6 million incident case of tuberculosis and 1.3 million deaths in 2012 [2]. Although one-third of population is infected by *Mycobacterium tuberculosis*, only 10% of those develop clinical disease during their lifetime. Multiple factors contribute to the risk of infection and development of tuberculosis including environmental factors, host–pathogen interactions and genetic factors [3]. Epidemiological studies indicate that the risk of developing tuberculosis in human is strongly influenced by genetic factors [4]. So far, gene polymorphisms of *SLC11A1* (formerly *NRAMP1*), vitamin D receptor, toll-like receptor 2, tumor necrosis factor-alpha and monocyte chemoattractant protein-1 have been identified to be associated with susceptibility of tuberculosis [5]–[9].

Human *P2X7* gene encoding the P2X7 receptor contains 13 exons and is localized on chromosome 12q24 [10]. P2X7 receptors are abundantly expressed in macrophages. *Mycobacterium tuberculosis* is a facultative intracellular pathogen and macrophages are the main reservoir for this agent. Activation of P2X7 by adenosine triphosphate (ATP) causes an immediate opening of a cation selective channel, permitting the influx of Ca^2+^ and Na^+^ and the efflux of K^+^. This process results the induction of the caspase cascade, apoptosis, and the activation of phospholipase D. Phospholipase D promotes phagosome–lysosome fusion and causes mycobacterial death [11]–[13].

Previous studies reported that various single-nucleotide polymorphisms (SNPs) in *P2X7* gene lead to the loss of receptor function, and the most common SNPs are the 1513A/C and -762T/C [14]. *P2X7* 1513 A/C gene polymorphism has been reported to be significantly associated with increased tuberculosis susceptibility [15]. However, the results from different studies on the association between the *P2X7* -762T/C (rs2393799) polymorphism and pulmonary tuberculosis susceptibility are conflicting [16]–[20]. Meta-analysis is a useful method for combining the results from different studies and providing more reliable conclusions [21]. Therefore, we performed a meta-analysis to investigate the association between the *P2X7*-762T/C gene polymorphism and the risk of pulmonary tuberculosis.

## Materials and Methods

### Literature Search Strategy

We conducted a systematic search on the PubMed, SCI, Elsevier, China National Knowledge Infrastructure (CNKI) and Wanfang Database (up to December 12, 2013). The following terms were used: (“tuberculosis” or : “TB”) and (“*P2X7*” or “*P2RX7*”) and (“polymorphism” or “mutation” or “variation”). We also searched the references of the retrieved literatures for additional studies. There was no restriction for time period, sample size, population, language, or types of reports. If more than one article was published using the same case series, only the study with largest sample size was included.

### Inclusion and Exclusion Criteria

Included studies in this meta-analysis met the following criteria: (a) a human case-control study on the association between *P2X7* -762T/C polymorphism and the risks of pulmonary tuberculosis; (b) containing available genotype data in cases and controls for estimating an odds ratio (OR) and 95% confidence interval (CI); (c) genotype distributions of control population were consistent with Hardy-Weinberg equilibrium (HWE). The exclusion criteria were: (a) reviews, letters, editorial articles and case reports; (b) studies on the association between other gene polymorphisms and pulmonary tuberculosis risks.

### Data Extraction

Two investigators (Yi and Cheng) extracted the data from all of the eligible publications according to the inclusion and exclusion criteria. Primary extraction data were reviewed by Zhen, and any disagreement was resolved by discussion among the authors. From each study, the following information was extracted: first author’s name, year of publication, study location, sample size, source of control, the genotyping method, the number of genotype frequencies in cases and controls.

### Statistical Analysis

The association between *P2X7* -762T/C polymorphism and pulmonary tuberculosis risks was estimated by calculating pooled ORs and 95%CI in the allele model (C vs. T), co-dominant model (CC vs. TT and CT vs. TT), dominant model (CC/CT vs. TT), and recessive model (CC vs. CT/TT). Significance of the pooled ORs was determined by Z test (p<0.05 was considered significant). Heterogeneity among included studies was checked by chi-square-based Q test and I^2^ test [22]. If the data showed no heterogeneity (p>0.05, I^2^<50%), Mantel–Haenszel fixed effects model was used, otherwise DerSimonian–Laird random effects model was used [23]. Hardy-Weinberg equilibrium (HWE) was also tested for included studies. Sensitivity analyses were performed by omitting certain studies each time. Publication bias was assessed by Begg’s adjusted rank correlation test and Egger’s regression asymmetry test.p<0.05 was considered as statistically significant [24]. All statistical analyses were performed using RevMan 5.0.23 (Cochrane Library Software, Oxford, UK) and STATA 11.0 Software (StataCorp LP, College Station, TX, USA).

## Results

### Studies Selection Process and Characteristics

**Figure 1:**
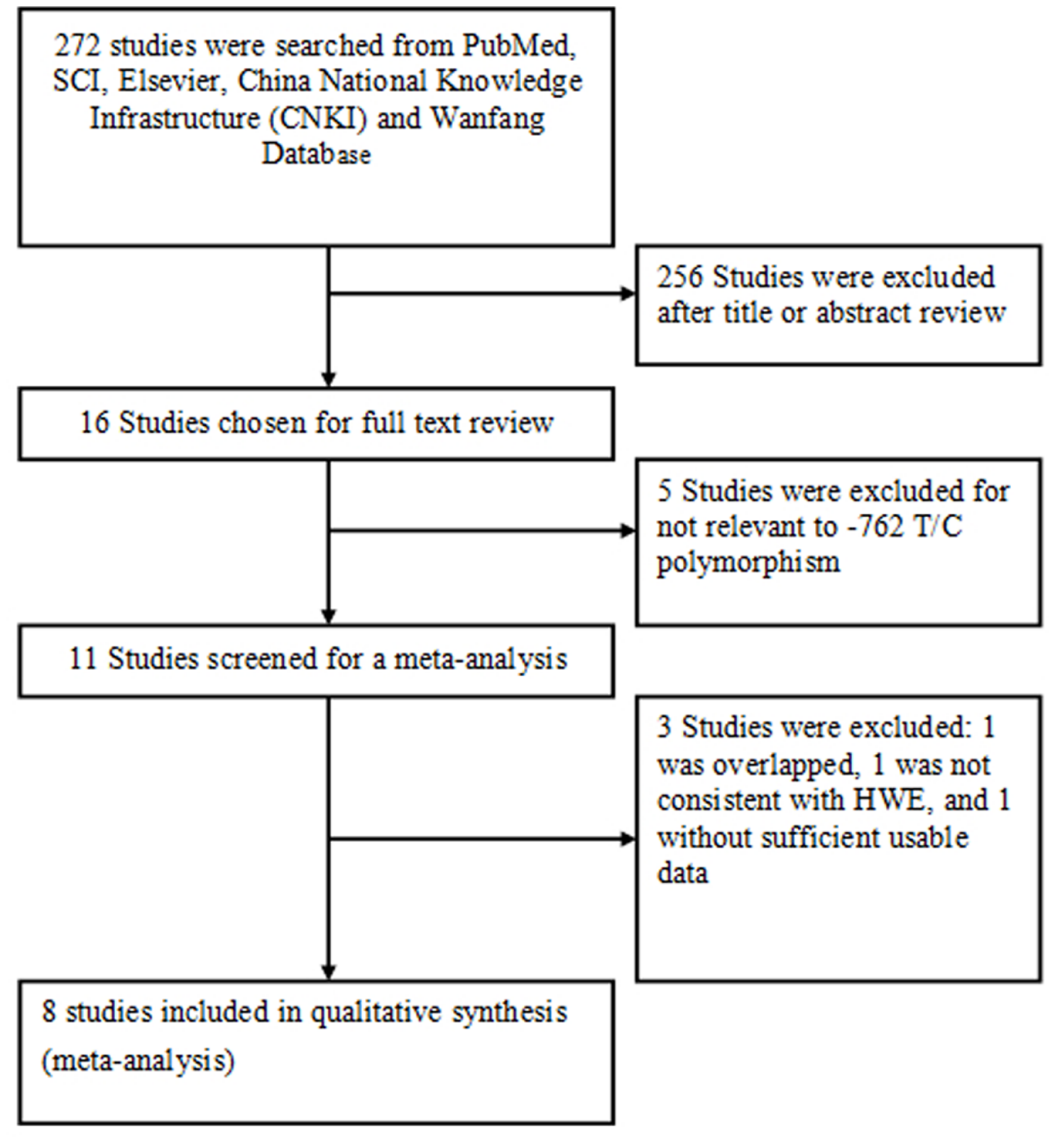
Flow diagram of the study selection.

A total of 272 studies were retrieved through database searching. After reading the titles and abstracts, 16 potential studies were identified for further investigation. Five studies were excluded for not being relevant to -762 T/C polymorphism. One study was excluded for data overlapping and one study was excluded for not reporting the usable data. One case-control study was excluded for being not consistent with HWE. Finally, 8 studies [16]–[18], [20], [25]–[28] were included for data extraction (Figure 1). Among these studies, 6 studies focused on the association between pulmonary tuberculosis risks and *P2X7* -762T/C polymorphism while other studies enrolled both pulmonary tuberculosis and extra-pulmonary tuberculosis patients. All of the 8 included studies were non-familial case-control studies. There was a total of 2207 subjects with pulmonary tuberculosis 1442 subjects were diagnosed by culture, 479 subjects were diagnosed by smear, and the methods of diagnosis of the rest subjects were not reported (Table 1). Genotype and allele distributions for each case-control study are shown in Table 2.

**Table 1:** Characteristics of the case-control studies included in the meta-analysis.

First Author	Year	Population	Genotypingmethod	Cases/Controls	MalePatients (%)	MaleControls (%)	Age ofcases	Age of controls	Diagnosis method	Control source
Bahari et al.	2013	Caucasian (Iran)	ARMS-PCR	150/150	38	34	48.97±21.2	45.36±16.1	Clinical symptoms,radiologic, smear, culture	Healthy individuals
Ben-Selma et al.	2011	African (Tunisia)	PCR-RFLP	168/150	75.6	90	44(14–78)	35(24–55)	Clinical examination,smear, culture	Healthy unrelated donors
Li et al.	2002	African (Gambia)	PCR-RFLP	323/347	67.4	100	34.7±13.2	30.3±7.5	Smear	Healthy unrelated donors
Mokrousov et al.	2008	Caucasian (Russia)	PCR-RFLP	190/127	47.7	64.7	31.7±11.1	32.2±12.0	Culture	Healthy unrelated donors
Nino-Moreno et al.	2007	Mixed (Mexico)	PCR-RFLP	92/110	50	DNR	46.1±17.6	32.4±12.2	Radiologic, culture	Healthy contacts
Sambasivan et al.	2010	Caucasian (India)	PCR-RFLP	156/100	50.7	57	30.4±18.3	35.6±13.3	Radiologic, smear	Healthy unrelated donors
Singla et al.	2012	Caucasian (India)	Allele-Specific PCR	286/392	63	55	33.1±15.4	36.4±14.9	DNR	Healthy unrelated individuals
Songane et al.	2012	Caucasian (Indonesia)	Sequencing	842/844	53.4	53.2	33	33	Clinical symptoms,radiologic, smear, culture	Healthy unrelated individuals

Abbreviations and definitions: ARMS PCR, Amplification Refractory Mutation System -Polymerase Chain Reaction; PCR-RFLP, Restriction Fragment Length Polymorphism analysis of PCR amplified fragments;

DNR: data not reported.

**Table 2:** Distributions of P2X7 -762T/C genotype and allele among TB patients and controls.

Author	Case			Control			Case		Control		HWE
	TT	TC	CC	TT	TC	CC	T	C	T	C	P value
Bahari et al.	25	54	71	6	40	104	104	196	52	248	0.39
Ben-Selma et al.	95	57	16	85	51	14	247	89	221	79	0.12
Li et al.	182	118	23	163	140	44	482	164	466	228	0.11
Mokrousov et al.	17	87	86	16	46	65	121	259	78	176	0.09
Nino-Moreno et al	52	32	8	51	44	15	136	48	146	74	0.27
Sambasivan et al.	30	88	38	36	49	15	148	164	121	79	0.8
Singla et al.	28	115	143	18	143	231	171	401	179	605	0.48
Songane et al.	248	413	181	255	412	177	909	775	922	766	0.65

### Quantitative Data Synthesis

**Figure 2:**
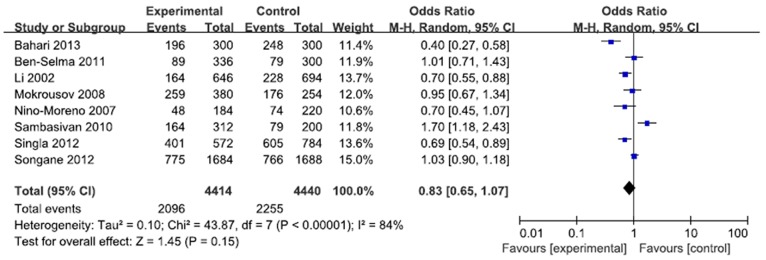
Forest plot and ORs with 95% CI of *P2X7* -762T/C polymorphism and pulmonary tuberculosis risk (C vs. T). The squares and horizontal lines correspond to the OR and 95% CI for each study. The area of the squares reflects the weight. The diamond represents the summary OR and 95% CI.

**Figure 3:**
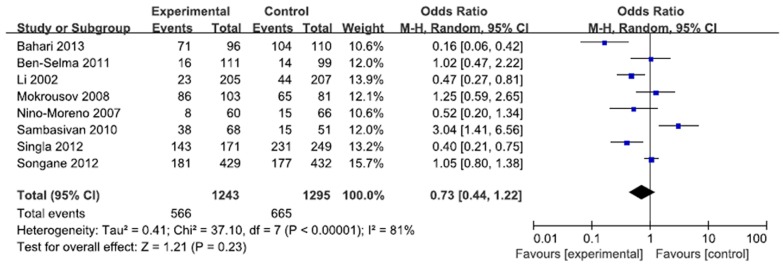
Forest plot and ORs with 95% CI of *P2X7* -762T/C polymorphism and pulmonary tuberculosis risk (CC vs. CT+TT).

**Figure 4:**
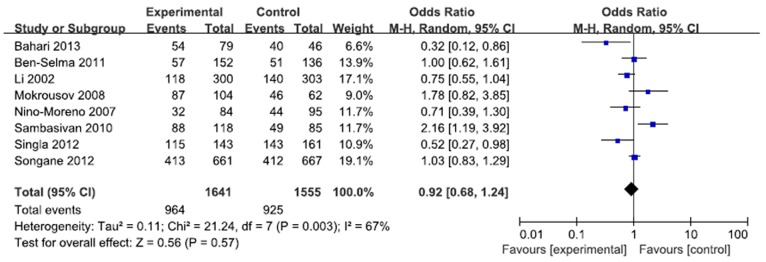
Forest plot and ORs with 95% CI of *P2X7* -762T/C polymorphism and pulmonary tuberculosis risk (CC vs. TT).

**Figure 5:**
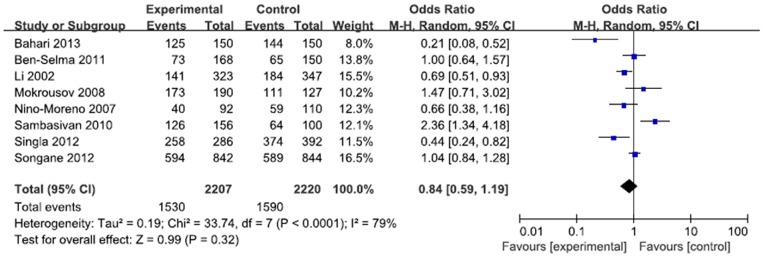
Forest plot and ORs with 95% CI of *P2X7* -762T/C polymorphism and pulmonary tuberculosis risk (CT vs. TT).

**Figure 6:**
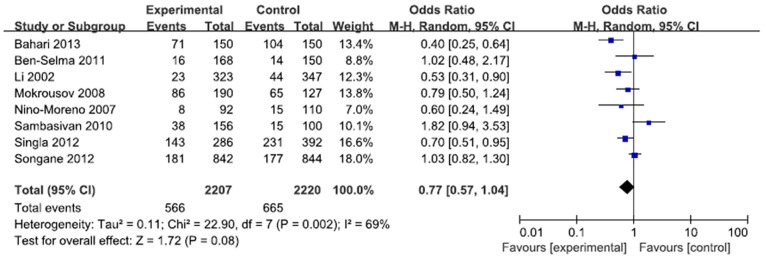
Forest plot and ORs with 95% CI of *P2X7* -762T/C polymorphism and pulmonary tuberculosis risk (CC+CT vs. TT).

A total of 2207 cases and 2220 controls in 8 case-control studies were pooled together for evaluation of the overall association between *P2X7* -762T/C polymorphism and risk of pulmonary tuberculosis. The pooled OR from all studies indicated no significant association between *P2X7* -762T/C polymorphism and pulmonary tuberculosis risk in allelic model (C vs. T: p = 0.15; OR = 0.83, 95% CI = 0.65–1.07) (Figure 2). There was no significant association in homozygous model (CC vs. TT: p = 0.23; OR = 0.73, 95% CI = 0.44 to 1.22) or heterozygous model (CT vs. TT: p = 0.57; OR = 0.92, 95% CI = 0.68 to 1.24) (Figure 3, 4). Similarly, dominant model (CC+CT vs. TT: p = 0.32; OR = 0.84, 95% CI = 0.59 to 1.19) and recessive model (CC vs. CT+TT: p = 0.08; OR = 0.77, 95% CI = 0.57 to 1.04) did not demonstrate any altered risk for pulmonary tuberculosis (Figure 5, 6).

Subgroup analysis by ethnicity did not detect any significant association between *P2X7*–762 T/C and pulmonary tuberculosis risk in Asians or Caucasians. A summary of results from all comparisons is listed in Table 3.

**Table 3:** Meta-analysis of *P2X7* gene -762T/C polymorphism and risk of pulmonary TB in each subgroup.

Category	C vs. T	CC vs. TT	CT vs. TT	CC+CT vs. TT	CC vs. CT+TT
	OR (95%CI), I^2^ (%)	OR (95%CI), I^2^ (%)	OR (95%CI), I^2^ (%)	OR (95%CI), I^2^ (%)	OR (95%CI), I^2^ (%)
Ethnicity					
Caucasian	0.86 (0.60–1.24), 89	0.79 (0.37–1.68), 87	0.97 (0.57–1.67), 77	0.85 (0.45–1.61), 86	0.81 (0.56–1.19), 79
African	0.82 (0.57–1.17), 66	0.66 (0.31–1.41), 62	0.82 (0.63–1.08), 0	0.80 (0.56–1.16), 48	0.69 (0.37–1.31), 50
Mixed	0.70 (0.45–1.07)	0.52 (0.20–1.34)	0.71 (0.39–1.30)	0.66 (0.38–1.16)	0.60 (0.24–1.49)
Overall	0.83 (0.65–1.07), 84	0.73 (0.44–1.22), 81	0.92 (0.68–1.24), 67	0.84 (0.59–1.19), 79	0.77 (0.57–1.04), 69

### Publication Bias

Begg’s funnel plot and Egger’s test were used to assess the publication bias of included studies. Publication bias was not observed in Begg’s funnel plot. The shape of the funnel plots was symmetrical and the Egger’s test did not show any evidence of publication bias. These data indicate that there is no significant publication bias in this meta-analysis.

### Test of Heterogeneity

Heterogeneity among studies in each model is shown in Table 3. The I^2^ showed a stable variation under all comparisons (C vs. T: I^2^ = 84%; CC vs. TT: I^2^ = 81%; CT vs. TT: I^2^ = 67%; CC vs. CT+TT: I^2^ = 79%; CC+CT vs. TT: I^2^ = 69%). Hence, the random effect model was applied to calculate the pooled ORs and 95%CI. In the subgroup analyses, p value for heterogeneity was not significant under heterozygous, dominant and recessive models of Asian population. However, there were heterogeneity under all comparisons in Caucasian population (Table 3).

### Sensitivity Analysis

Sensitivity analysis was performed to assess the influence of each individual study on the pooled OR by deleting one single study each time. The results showed that no individual study affected the pooled OR significantly, indicating the stability of this meta-analysis.

## Discussion

In the present meta-analysis, we show that *P2X7* gene -762T/C polymorphism is not associated with the risk of pulmonary tuberculosis. Subgroup analysis by ethnicity did not detect any significant association between *P2X7*–762 T/C and pulmonary tuberculosis risk in Asians or Caucasians.

Our meta-analysis included a total of 8 case-control studies with 2207 cases and 2220 controls, which is substantially larger than a previous meta-analysis on *P2X7* gene -762T/C polymorphism and pulmonary tuberculosis risk [15]. We performed the comparisons of all genetic models in this meta-analysis, while only allelic frequencies were compared in the previous meta-analysis [15]. The conclusion of our meta-analysis that *P2X7* gene -762T/C polymorphism is not associated with pulmonary tuberculosis risk is consistent with the previous meta-analysis [15].

The polymorphisms in the promoter or coding region of *P2X7* gene may alter its expression or function. However, the -762T/C polymorphism of *P2X7* does not appear to be related with altered receptor expression [16]–[18]. Li and colleagues found that patients with active tuberculosis exhibited reduced *P2X7* receptor expression levels on mononuclear cells which were recovered during chemotherapy. This suggested that the expression of *P2X7* receptor may be regulated by host- or pathogen-generated inhibitory factors [16], [25], [26].

Heterogeneity is a potential problem that might affect the interpretation of the results. Therefore, the control group should conform to HWE to ensure similarity of the population genetic background and the reliability of association analyses. One of the potentially eligible studies did not conform to HWE and was excluded [19]. The heterogeneity could also be caused by ethnic variations, environmental factors and methodological factors in study design. The leave-one-out sensitivity analysis did not affect the results of this pooled analysis, indicating that our results were reliable. There was no publication bias for the association between *P2X7*–762 T/C polymorphism and pulmonary tuberculosis risk.

The present meta-analysis had several limitations. First, we only included the studies published in English and Chinese. Some related studies published in other languages might be missed in our meta-analysis. Second, although the funnel plot and Egger’s test did not show any publication bias, the influence of bias in the present analysis could not be completely excluded. A more precise analysis could be performed if more detailed data such as age, sex and exposure of each subject was available.

In conclusion, this meta-analysis indicates that *P2X7* -762T/C polymorphism is not associated with pulmonary tuberculosis risk. Further studies with more stringent design and larger sample size are required to validate this conclusion.

## Supporting Information

Checklist S1 **PRISMA Checklist.**
(DOC)
