# Correction: A Meta-Analysis of *P2X7* Gene-762T/C Polymorphism and Pulmonary Tuberculosis Susceptibility

**DOI:** 10.1371/journal.pone.0121649

**Published:** 2015-03-31

**Authors:** The PLOS ONE Staff

There are errors in the legends for Figs. 3–6. The complete, correct figure legends for Figs. 3–6 are:

**Fig 3:**
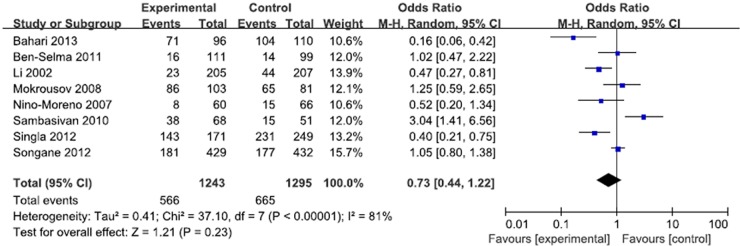
Forest plot and ORs with 95% CI of *P2X7*–762T/C polymorphism and pulmonary tuberculosis risk (CC vs. TT).

**Fig 4:**
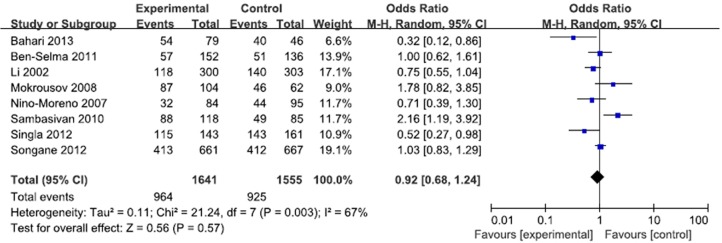
Forest plot and ORs with 95% CI of *P2X7*–762T/C polymorphism and pulmonary tuberculosis risk (CT vs. TT).

**Fig 5:**
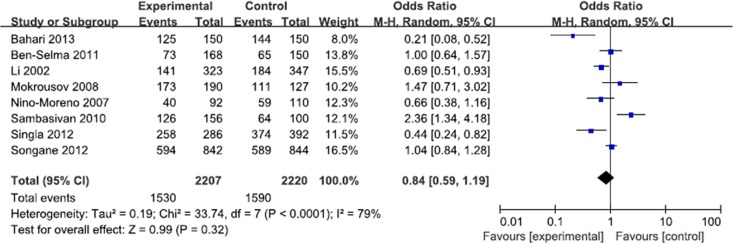
Forest plot and ORs with 95% CI of *P2X7*–762T/C polymorphism and pulmonary tuberculosis risk (CC+CT vs. TT).

**Fig 6:**
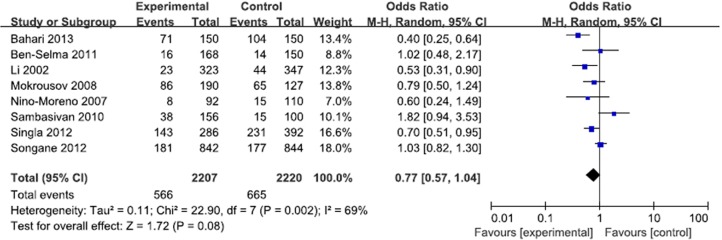
Forest plot and ORs with 95% CI of *P2X7*–762T/C polymorphism and pulmonary tuberculosis risk (CC vs. CT+TT).

There are errors in Tables 1 and 3. Please see the corrected Tables 1 and 3 here.

**Table 1:** Characteristics of the case-control studies included in the meta-analysis.

First Author	Year	Population	Genotyping method	Cases/Controls	Male Patients (%)	Male Controls (%)	Age of cases	Age of controls	Diagnosis method	Control source
Bahari et al.	2013	Caucasian (Iran)	ARMS-PCR	150/150	38	34	48.97±21.2	45.36 ± 16.1	Clinical symptoms, radiologic, smear, culture	Healthy individuals
Ben-Selma et al.	2011	African (Tunisia)	PCR-RFLP	168/150	75.6	90	44(14–78)	35(24–55)	Clinical examination, smear, culture	Healthy unrelated donors
Li et al.	2002	African (Gambia)	PCR-RFLP	323/347	67.4	100	34.7±13.2	30.3±7.5	Smear	Healthy unrelated donors
Mokrousov et al.	2008	Caucasian (Russia)	PCR-RFLP	190/127	47.7	64.7	31.7±11.1	32.2±12.0	Culture	Healthy unrelated donors
Nino-Moreno et al.	2007	Mixed (Mexico)	PCR-RFLP	92/110	50	DNR	46.1±17.6	32.4±12.2	Radiologic, culture	Healthy contacts
Sambasivan et al.	2010	Asian (India)	PCR-RFLP	156/100	50.7	57	30.4±18.3	35.6±13.3	Radiologic, smear	Healthy unrelated donors
Singla et al.	2012	Asian (India)	Allele-Specific PCR	286/392	63	55	33.1± 15.4	36.4 ± 14.9	DNR	Healthy unrelated individuals
Songane et al.	2012	Asian (Indonesia)	Sequencing	842/844	53.4	53.2	33	33	Clinical symptoms, radiologic, smear, culture	Healthy unrelated individuals

Abbreviations and definitions: ARMS PCR, Amplification Refractory Mutation System-Polymerase Chain Reaction; PCR-RFLP, Restriction Fragment Length Polymorphism analysis of PCR amplified fragments;

DNR: data not reported.

**Table 3:** Meta-analysis of *P2X7* gene-762T/C polymorphism and risk of pulmonary TB in each subgroup.

Category	C vs. T	CC vs. TT	CT vs. TT	CC+CT vs. TT	CC vs. CT+TT
	OR (95%CI), I^2^ (%)	OR (95%CI), I^2^ (%)	OR (95%CI), I^2^ (%)	OR (95%CI), I^2^ (%)	OR (95%CI), I^2^ (%)
Ethnicity					
CaucasianAsian	0.61 (0.26–1.45), 911.04 (0.70–1.55), 88	0.46 (0.06–3.39), 911.06 (0.43–2.59), 88	0.78 (0.15–4.17), 861.05 (0.56–1.98), 81	0.56 (0.08–3.87), 911.04 (0.50–2.15), 87	0.56 (0.29–1.10), 761.01 (0.66–1.53), 75
African	0.82 (0.57–1.17), 66	0.66 (0.31–1.41), 62	0.82 (0.63–1.08), 0	0.80 (0.56–1.16), 48	0.69 (0.37–1.31), 50
Mixed	0.70 (0.45–1.07)	0.52 (0.20–1.34)	0.71 (0.39–1.30)	0.66 (0.38–1.16)	0.60 (0.24–1.49)
Overall	0.83 (0.65–1.07), 84	0.73 (0.44–1.22), 81	0.92 (0.68–1.24), 67	0.84 (0.59–1.19), 79	0.77 (0.57–1.04), 69
